# How Useful Is Image-Based Active Learning for Plant Organ Segmentation?

**DOI:** 10.34133/2022/9795275

**Published:** 2022-02-24

**Authors:** Shivangana Rawat, Akshay L. Chandra, Sai Vikas Desai, Vineeth N. Balasubramanian, Seishi Ninomiya, Wei Guo

**Affiliations:** ^1^Department of Computer Science and Engineering, Indian Institute of Technology, Hyderabad, India; ^2^Department of Computer Science, University of Freiburg, Germany; ^3^Graduate School of Agricultural and Life Sciences, The University of Tokyo, Japan

## Abstract

Training deep learning models typically requires a huge amount of labeled data which is expensive to acquire, especially in dense prediction tasks such as semantic segmentation. Moreover, plant phenotyping datasets pose additional challenges of heavy occlusion and varied lighting conditions which makes annotations more time-consuming to obtain. Active learning helps in reducing the annotation cost by selecting samples for labeling which are most informative to the model, thus improving model performance with fewer annotations. Active learning for semantic segmentation has been well studied on datasets such as PASCAL VOC and Cityscapes. However, its effectiveness on plant datasets has not received much importance. To bridge this gap, we empirically study and benchmark the effectiveness of four uncertainty-based active learning strategies on three natural plant organ segmentation datasets. We also study their behaviour in response to variations in training configurations in terms of augmentations used, the scale of training images, active learning batch sizes, and train-validation set splits.

## 1. Introduction

Deep learning models have been widely used for various plant phenotyping tasks by formulating them as standard vision tasks such as image classification [1–4], object detection [5–8], and semantic segmentation [9–11]. In this work, we focus on the task of semantic segmentation, a dense prediction task where the goal is to predict a class label for every pixel in an image. Plant phenotyping researchers have used the segmentation task in a variety of ways, ranging from simpler tasks of separating plants from their backgrounds [12] to more challenging tasks of tracking plant growth and yield such as individual leaf segmentation [13], leaf counting [9], estimating plant stem pose [14], and detecting diseases [15].

While semantic segmentation has many applications in plant phenotyping, training semantic segmentation models requires dense pixel-wise annotations which is labour-intensive, time-consuming, and often requires domain experts spending hundreds of hours on only labeling and crowdsourcing [13, 16]. Plant phenotyping datasets add another layer of complexity to the data annotation process owing to factors such as varied lighting conditions, heavy occlusion, camouflaged foreground objects, and variability in size, shape, and pose [13] Active learning (AL), a subfield of machine learning, helps reduce the annotation burden by selecting and labeling only the most *informative samples* [17]. AL sampling techniques reasonably alleviate the bottleneck of data annotation which is particularly desirable in the context of training deep neural networks. Owing to the success of data-driven learning methods, many practitioners have relied on collecting, labeling, and maintaining large amounts of data to solve tasks of their interest. This popularized pool-based variant of AL methods which specifically allow for selecting *active samples* from a large pool of unlabeled data.

AL for semantic segmentation has been well-studied on standard datasets such as PASCAL VOC [18] and Cityscapes [16]. In the context of plant phenotyping, there has been encouraging evidence of AL's effectiveness on classification and detection based tasks [19, 20]. However, AL's effectiveness on plant-based segmentation datasets has not received much attention in the literature. In this paper, we bridge this gap by empirically studying and benchmarking the effectiveness of four popular existing AL techniques on three plant segmentation datasets. AL methods in this study are performed at image-level, i.e., a queried image is labeled entirely before being added to the labeled pool, hence the name *image-based active learning*. Alternatively, some recent AL works [21–23] reported great model performances even while actively labeling only a small portion of images, we leave evaluation of these methods to future works. In this work, we study how the AL techniques respond to changes in the training configurations such as with/without data augmentation, effect of image scale, initial labeled pool size, batch size, and train validation split. We feel such a detailed study would allow the researchers and practitioners to understand how to integrate AL into their plant phenotyping pipeline to reduce the annotation burden. To the best of our knowledge, this is the first work that discusses the effectiveness of AL in plant organ segmentation in RGB images. In this work, our aim is to investigate how well the benefits of AL transfer from classification to semantic segmentation, which is a more complex prediction task. To that end, we have chosen three diverse datasets for evaluation that vary in terms of image resolution, lighting conditions, and task complexity in general.

The paper is organized as follows.  Section 2 describes the related work.  Section 3 introduces the pool based active learning setting in the context of semantic segmentation and discusses the query strategies.  Section 4 describes the datasets used and experimental setup. In Sections 5 and 6, we report our observations, discuss our results, and make concluding statements, respectively.

## 2. Related Work

### 2.1. Semantic Segmentation in Plant Phenotyping

Prior to deep learning, plant researchers relied on traditional image processing techniques such as edge detection, thresholding, graph partitioning, and clustering [24] to obtain segmentation maps of plant organs [25–27]. With the rapid growth and success of deep neural networks, intelligent model-based automatic segmentation of plant organs has now become an unavoidable prerequisite for measuring more complex phenotypic traits. Aich and Stavness [9] perform leaf counting by training two separate models—a segmentation model to generate leaf segmentation maps and a regression model that takes the segmented maps as input to perform counting. Choudhury et al. [14] introduced an algorithm that uses plant segmentation masks to compute stem angle, a potential measure for plants' susceptibility to lodging. Ma et al. [15] achieved robust disease segmentation in greenhouse vegetable foliar disease symptom images. They proposed a decision tree-based two-step coarse-to-fine segmentation method. Shi et al. [12] proposed a multiview approach that maps 2D segmentation maps to 3D point clouds on a multiview tomato seedling dataset to increase prediction accuracy. We refer the readers to Li et al. [28] for a more comprehensive review of the applications of semantic segmentation in plant phenotyping.

### 2.2. Active Learning for Plant Phenotyping

The key hypothesis of AL is that if the learning algorithm is allowed to choose the data from which it learns, it will perform better with less training. AL techniques have long been used for reducing annotation effort [17, 29–33]. However, only a handful of works have been published that apply AL on plant phenotyping tasks. In the context of robotic plant phenotyping, Kumar et al. [34] proposed a Gaussian process-based AL algorithm to enable an autonomous system to collect the most informative samples in order to accurately learn the distribution of phenotypes in the field. Grimm et al. [35] proposed a model-free approach to plant species classification with the help of AL. More recently, Nagasubramanian et al. [19] comprehensively studied the usefulness of AL in plant phenotyping on two classification datasets and showed that AL techniques outperform random sampling and indeed reasonably reduce labeling costs. For object detection task, Chandra et al. [20] achieved superior model performance compared to random sampling while saving over 50% of annotation time on sorghum-head and wheat-panicle detection datasets by exploiting weak supervision for obtaining informative samples.

## 3. Active Learning for Semantic Segmentation

In this section, we first describe the widely used pool-based active learning setting. Subsequently, we describe the AL query methods for semantic segmentation which are evaluated in our experiments.

### 3.1. Pool-Based Active Learning

Pool-based AL assumes that there exists a large collection of unlabeled data to solve the learning problem at hand. In this data-centric deep learning era, it is a fair assumption to make. We consider a training set of images *T* which is a union of the unlabeled set *U* and the labeled set *L*. Initially, *L* is empty and *U* contains the entire dataset, because initially, all data is unlabeled. We first randomly sample a small subset of images from *U*, label them, and then, move those images from *U* to *L*. Now, this initial labeled pool *L* is used to train an initial segmentation model *M*. From here, we run multiple AL cycles. In each cycle, we intelligently sample a batch of images from *U*, label them, and move them to *L*, which is used to retrain model *M*. For intelligent sampling, we use a query function *Q*(*M*, *U*) which takes the current model *M* and the current unlabeled set *U* as input. Based on model *M*'s predictions on each image in *U*, the query function *Q* calculates an informativeness score for each image and selects a batch of *b* most informative images from *U*. This cycle repeats until either the sampling budget is exhausted or until the model achieves a satisfying test set performance. This framework is described in Algorithm 1.

### 3.2. Query Strategies

We obtain an informativeness score for every image by averaging the pixel-wise informativeness scores calculated by the AL query methods. Formally, the informativeness score *S*_*I*_ of an image *I* is given by

(1) $$\begin{array}{c} S_{I}=\frac{1}{\;|\;H*W\;|\;}\underset{i\leq H,j\leq W}{\sum }S_{\left(i,j\right)}. \end{array}$$

Here, *S*_(*i*, *j*)_ is the informativeness score of (*i*, *j*) pixel, and *H* and *W* are the height and width of the image *I*, respectively. Further, we select the samples which have the maximum informativeness score based on cycle budget size (i.e., top *k* images with highest *S*_*I*_).

We now describe the informativeness scores used as active learning query strategies in our experiments. We decided to evaluate the following AL methods in this study because while they are easy to implement and experiment with, they are also some of the best baselines on CamVid and Cityscapes segmentation datasets, especially Entropy (Section 3.2.3) [21–23]. Apart from the following query methods, we also perform random (RAND) sampling, which serves as a baseline.

#### 3.2.1. Least Confidence (LC)

Least confidence method [36] queries instances which the model is least certain about. This approach is often straightforward for models which provide prediction probabilities. For example, in the case of binary classification, this method simply queries the instances whose posterior probability of being positive is nearest to 0.5 [17]. A more general variant is as follows:

(2) $$\begin{array}{c} S_{\left(i,j\right)}=1-p, \end{array}$$

where *p* is the trained model's maximum prediction probability after applying Softmax, i.e., the probability of pixel (*i*, *j*) being a nonbackground object, in the context of semantic segmentation.

#### 3.2.2. Margin (MAR)

The criterion for the least confidence strategy only considers information about the most probable label. Thus, it effectively discards information about the rest of pixel's probability distribution. To overcome this, Scheffer et al. [37] proposed a multiclass sampling variant. Here, for each pixel, we make use of model's probabilities of both first (*p*_1_) and second (*p*_2_) most probable class labels. The informativeness score for a pixel at (*i*, *j*) is given by

(3) $$\begin{array}{c} S_{\left(i,j\right)}=-\left(p_{1}-p_{2}\right). \end{array}$$

Intuitively speaking, samples with large margins are easy since the trained model has little confusion in discriminating between the two most likely class labels. Here, *p*_1_ − *p*_2_ is the margin, and the negative sign is used here only for convenience since less margin indicates more informativeness. Thus, we can select samples with maximum *S*_*I*_, consistent with other query methods.

#### 3.2.3. Entropy (ENT)

Margin sampling still ignores most of the output distribution when dealing with large label sets. A more general uncertainty sampling method (and perhaps the most popular one) is Shannon's Entropy [38] as an informativeness measure. This method makes use of all model's predicted probabilities for a given pixel. The informativeness score is given by

(4) $$\begin{array}{c} S_{\left(i,j\right)}=-\underset{c\in \left\{1\cdots C\right\}}{\sum }\;p_{c}\log p_{c}, \end{array}$$

where *C* is the number of classes in the dataset and *p*_*i*_ is the probability score of the corresponding class. Then, *S*_*I*_ is calculated using Equation (1), and the samples with the maximum value of *S*_*I*_ are selected.

#### 3.2.4. Deep Bayesian Active Learning (DBAL)

Deep Bayesian active learning by Gal et al. [29] trains model *M* with dropout layers to simulate Monte-Carlo sampling. At inference time, the dropout layers are applied, and inference is done *T* times. The probability scores for the *T* dropout runs are averaged, and entropy is computed as follows:

(5) $$\begin{array}{c} S_{\left(i,j\right)}=-\underset{c\in \left\{1\cdots C\right\}}{\sum }\left(\frac{1}{T}\underset{t}{\sum }\;p_{t}^{c}\right)\log\left(\frac{1}{T}\underset{t}{\sum }\;p_{t}^{c}\right), \end{array}$$

where *C* is the number of classes in the dataset and *p*_*t*_^*c*^ is the probability of the class *c* for the dropout run *t*. This query selection method is also known as the query-by-committee algorithm [39].

## 4. Experimental Settings

### 4.1. Datasets

*ACFR Orchard Fruit Dataset* (Apple) dataset by Bargoti and Underwood [40] contains 1120 RGB images of apples and their pixel-level labels. All the images were captured at a resolution of 202 × 308. They are noisy and have poor lighting accompanied with leaf occlusions. The dataset contains some images which do not contain any apples, we removed those images as a preprocessing step. This left us with 1081 images; we further cropped the images to a resolution of 200 × 300. Finally, we divided the dataset into train, validation, and test sets. We split the dataset with 681 images in the train set, 100 images in the validation set, and 300 images in the test set.

*UTokyo_Wheat_2020* (Wheat) dataset is a subset obtained from the work of David et al. [41], containing 2674 RGB images. Since the original dataset did not have segmentation labels, we labeled it with polygons for this study. Originally, the images were captured at resolution 1400 × 4200 roughly. The dataset consists of images which were captured in reasonably good lighting conditions containing wheat ears which have a different texture compared to the background. We resized all the images to a fixed size of 1024 × 400 and sliced (https://image-bbox-slicer.readthedocs.io/) them into patches of size 512 × 400. We obtained 5348 patches of size 512 × 400; out of which, we discarded the ones which did not contain any wheat ears. Finally, we were left with 3547 images which we divide into the train, validation, and test sets. We split the dataset with 2047 images in the train set, 500 images in the validation set, and 1000 images in the test set.

*UTokyo_Rice_2013* (Rice) dataset by Desai et al. [5] contains 1953 RGB images of size 1296 × 864. Similar to the Wheat dataset, the original Rice dataset did not have segmentation labels so we labeled the datasets with polygons for this study. We resized all the images to a size of 648 × 432 and then cropped them to obtain images of size 640 × 432. The dataset is then split into a train set of size 1203 images, validation set with 250 images, and test set with 500 images.

**Figure 1:**
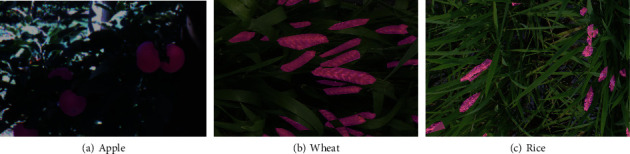
Example images for the three datasets with foreground masks overlayed. Segmentation labels are shown in magenta-colored overlaps.

While the train and validation set splits are done randomly at the beginning of each experiment, the test set is constant across all the reported experiments. Example images from all three datasets and their corresponding segmentation maps are shown in Figure 1 for reference.

### 4.2. Model Architecture and Hyperparameters

**Figure 2:**
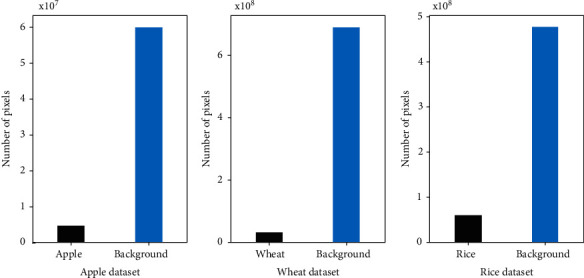
Class imbalance in the three datasets.

We use Deeplabv3+ [42] as our segmentation model with a ResNet-50 [43] backbone (https://github.com/yassouali/pytorch-segmentation). Since the DBAL method requires dropout [44], we add it to the backbone with a probability of 0.1. For consistency in the experimental setting, we used the dropout-enabled backbone for training in the case of all AL methods. In the case of DBAL method, we aggregated model inferences over *T* = 25 dropout runs (stochastic forward passes). Only one model inference is considered for all other AL methods. The ResNet backbone is initialized with ImageNet pretrained weights. We opted to use focal loss [45] because the datasets are highly imbalanced, as shown in Figure 2.

In all our experiments, we use the Adam optimizer with a constant learning rate. For the Apple and the Wheat dataset, the learning rate is set to 0.0001, whereas for the Rice dataset, the learning rate is set to 0.001. In every AL cycle, we retrain the model on the updated labeled pool for 50 epochs. Note that we initialize the model with ImageNet pretrained weights in every AL cycle, before training on the labeled pool. The model with the best performance (IoU) on the validation set is used for active sampling. To account for randomness, we repeat each experiment 3 times and report the mean and standard deviations of IoU. In case of the Apple dataset, we populate the initial pool with 50 images. We use 100 images in the initial pool of Wheat and Rice dataset. We set the AL batch size same as that of initial pool size for all three datasets. Throughout the paper, we report the intersection over union (IoU) of the foreground class as the evaluation metric.

## 5. Results and Discussion

### 5.1. Active Learning Performance

**Figure 3:**
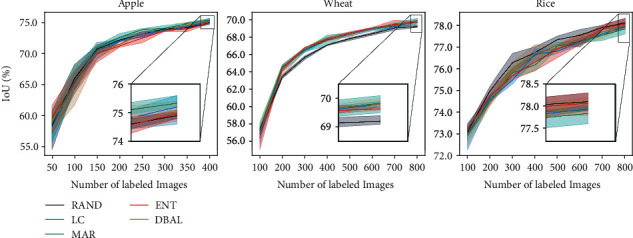
Active learning performance on Apple, Wheat, and Rice datasets.

**Figure 4:**
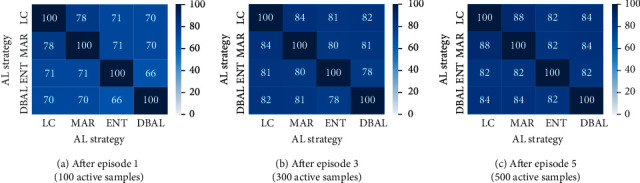
Overlap between active sample sets picked by different AL methods (Wheat).

We first study the performance of the four AL methods with respect to random sampling on the three datasets. We observe in Figure 3 that AL strategies do not show a consistent improvement across all datasets. For the Apple and the Wheat dataset, we see an IOU improvement of 0.43% and 0.53% over RAND, respectively, using MAR. However, for the Rice dataset, RAND consistently performs better than all the active learning techniques. We suspect that model's uncertainty on Rice images is overestimated due to the very nature of the Rice dataset, i.e., lack of clear visual discrimination between background and foreground pixels. While being very popular in the vision community for their performance gains, we see little to no benefit from ENT and DBAL methods on all three datasets. Overall, there was no clear winner. To understand this further, we took a closer look at the sample sets actively sampled by the AL methods. As an ablation study, we used a model trained on a randomly chosen initial labeled pool of Wheat and sampled unlabeled data points based on the informativeness scores of all the AL methods (with replacement). We noticed (see Figure 4) significant overlap between actively sampled sets across AL methods. The overlap increased even more in later stages of AL cycles, and this was consistent across all three datasets. We believe this is the case due to the similarity (or proportionality) between informativeness scores calculated by the query metrics at hand, largely contributed by the binary nature of the task, which is clearly explained in Chapter 3 of [17]. Apple and Rice overlap statistics are reported in the supplementary in Figures S1 and S2, respectively.

While it is understood why there was no clear winner amongst the AL methods, we suspect that the AL methods are underperforming (compared to random sampling) due to the imbalanced nature of the datasets. While the issue of class imbalance is common in segmentation datasets [16, 18], it is particularly exacerbated in plant phenotyping datasets where the pixel counts of foreground objects are significantly lower in comparison. Figure 2 shows the pixel counts of foreground objects and background of the three datasets. Is image-level average (see Equation (1)) a bad approximation for capturing model's uncertainty over these high class-imbalanced images? Perhaps a balancing factor could be added to the existing AL methods to dampen the contribution of the pixels that are most likely to be background towards the image informativeness score. This is, however, out of the scope of this work, and it would be an interesting direction that merits further investigation.

### 5.2. Differing Experimental Conditions

Next, we study the impact of changes in experimental conditions such as data augmentations, image scales, initial labeled pool size, AL batch size, validation set size, and train-val split ratio on AL methods and model performance in general.

#### 5.2.1. Image Scale

**Figure 5:**
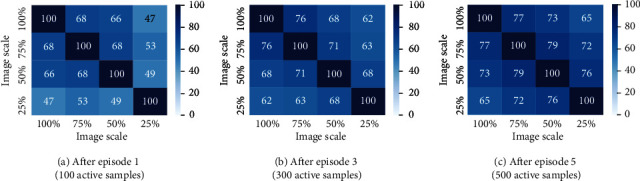
Overlap between active sample sets picked by ENT models trained on different image scales (Wheat).

Image resolution plays a crucial role while training deep neural networks. Images with high resolution capture a lot of detail and are helpful in training highly accurate deep learning models but at the cost of computational overhead. On the other hand, images with low resolution are faster to process and train and help us in saving a lot of computation cost. We try to address the trade-off between computation cost and annotation cost. We first study the effect of scale on model performance. We experiment with four different image scales 25%, 50%, and 75% of original image size and also at 100% scale. The AL cycles are then carried out at said four scaled versions of the images. Post five AL cycles, we checked the overlap between active sample sets picked by models at all four scales (excluding the randomly chosen initial labeled pools). After five episodes of AL in all four scales, we checked for overlap between active sample sets collected across the scales. Precisely, we calculate percentage of overlap between respective actively sampled sets from 25%, 50%, 75%, and 100% scales after episodes 1, 3, and 5. The overlap results for the ENT AL method on the Wheat dataset are shown in Figure 5, averaged over 3 runs. We observe significantly high overlap between the sample sets. After just one episode, the highest overlap of 68% is observed between 100% and 75% scale sets and about 50% overlap between 100% and 25% sets. This overlap increases much more in later AL episodes. This suggests that, in the context of AL, one can end up with largely similarly labeled datasets even when operating at smaller image scales.

**Figure 6:**
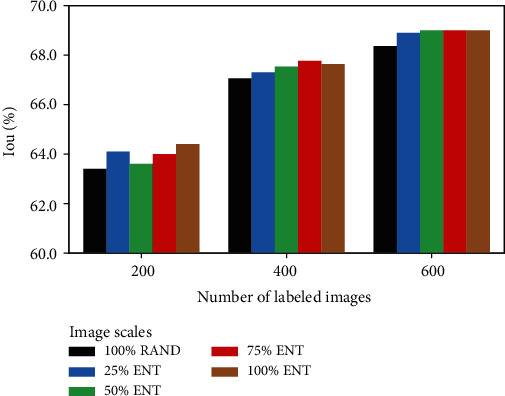
Performances of models trained on active sample sets from different image scale configurations (Wheat).

We tested if active samples transfer well from smaller image scales, and indeed, they did. To that end, we used the ENT active sample sets picked by models trained on smaller image scales, resized them back to the original scale, and trained a model until convergence on the full-resolution images. All results were averaged over 3 runs. As seen in Figure 6, we observe similar performance by all active sample sets compared to the 100% active sample sets at multiple stages of the AL cycles. Moreover, all three (25%, 50%, and 75%) sets perform better than 100% RAND sample sets which confirms that the *activeness* of the sample sets is still preserved after resizing back to original. Similar overlap statistics on Apple and Rice datasets in case of the ENT method are reported in Figures S3 and S4, respectively, of supplementary. Operating in smaller image scales would mean that the models have to work with smaller objects. This typically makes both model training and inference more challenging [46]. We observe this clearly in the Rice dataset. The Rice dataset, which has comparatively smallest objects (w.r.t. image size) among the three datasets, has the highest average active sample overlap after five AL cycles (shown in Figure S3). On the surface, this subtly implies that the role of object scale is trivial for AL methods. However, we must observe in Figure 3 that all AL methods perform worse than RAND on Rice dataset, emphasizing that object scale indeed plays a crucial role in model training, inference, and subsequently, on AL performance as well. A further investigation into how AL methods respond to varying object scales is warranted.

#### 5.2.2. Data Augmentation

Automatic plant phenotyping is hard and complicated. Collection of raw data from fields and greenhouses is a complicated task owing to factors such as vast crop varieties and phenotype diversities and heavy reliance on growth seasons, climate changes, and more. This process is further complicated when specific learning tasks require a large amount of labeled data for training. Adding augmented data to the labeled pool is a well-established method that is known to contribute to model's better generalization. To that end, we examine if data augmentation helps the segmentation model and the AL methods. We repeated the AL experiments with the following augmentations: scaling, rotation, and horizontal flipping. In case of the Wheat dataset, model performance improved by 1.166% IoU. We observe that simple data augmentations do not contribute much in the case of Apple and Rice datasets, showing a mere performance increase of 0.1% and 0.13% IoU, respectively (see Table 1).

Nearly all the AL methods performed slightly better than their unaugumented variants. But this is also seen in the case of RAND. This suggests that there is a need for more task-specific, tailored, organ augmentation techniques, similar to [47] which better simulate environmental occlusions, etc. Such techniques could make the segmentation models more robust and likely make them better uncertainty estimators for AL methods to utilize.

#### 5.2.3. Initial Labeled Pool Size

**Figure 7:**
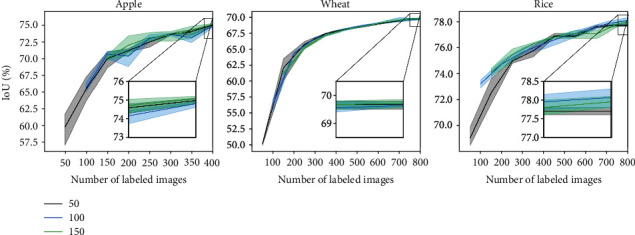
Varying initial pool size.

The initial pool provides a good initialization for the model so that it can be used to obtain uncertainty estimations over the unlabeled pool. The model trained on the initial pool is responsible for selecting samples for further AL cycles. To study the effect of the size of the initial pool on the performance of each active learning strategy, we experiment with initial pool sizes of 50, 100, and 150. The results are shown in Figure 7. We observe that performance gain due to different initial pool sizes is dataset dependent. While the rice segmentation model is initially benefited by a larger initial pool size, the models on other datasets do not show any significant improvements. Interestingly, the performance difference between models started with different initial pools vanishes in the later AL cycles. We report results of this experiment on all other AL methods in Figure S5 of supplementary.

#### 5.2.4. Active Learning Batch Size

**Figure 8:**
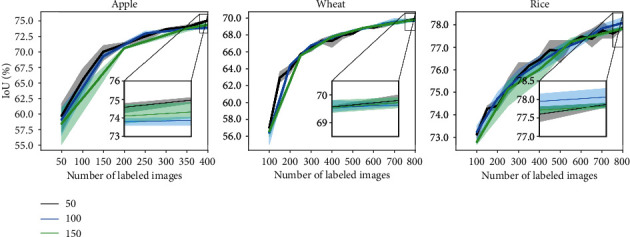
Varying AL batch size.

We now explore the effect of different AL batch sizes on AL methods as small batch sizes could select samples which are not diverse. On the other hand, large batch sizes might select samples which are redundant and not very informative to the model. We experiment with 3 different batch sizes; for all datasets, we experiment with batch sizes of 50, 100, and 150. The results for ENT on all datasets are shown in Figure 8. All the other sampling strategies observe similar trends. The results for all the other AL sampling strategies are given in Figure S6. We observe that there are slight variations in the initial AL cycles but that performance difference vanishes for higher AL cycles.

#### 5.2.5. Train Validation Split

**Figure 9:**
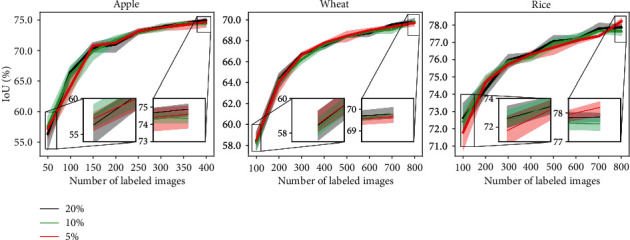
Varying validation set size.

In each episode of AL, the best model is saved based on validation set accuracy. The validation set used for saving the best model should also be labeled. This annotation cost is mostly ignored in the literature. We study the effect of the size of the validation set on different AL strategies. We keep the training set (*T*_*r*_) as constant and sample subsets from the original validation set (*V*) to obtain smaller validation sets and experiment with those. We choose the subsets of size equal to 5%, 10%, and 20% of the set (*T*_*r*_ + *V*). We show the results for ENT sampling for all the datasets in Figure 9. We observe that the size of the validation set does not have much effect on the model performance in the long run, although extremely small validation sets are not appreciated as it could lead to overfitting.

**Figure 10:**
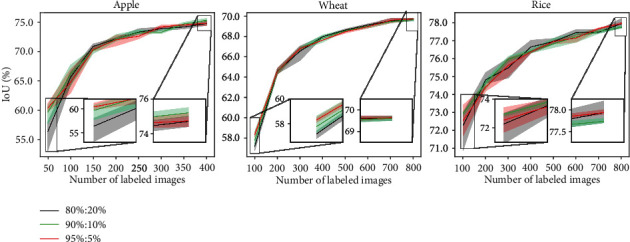
Varying train-val split ratio.

Next, we try to explore the trade-off among the size of the train and validation set. We experiment with train-val splits of ratio 80% : 20%, 90% : 10%, and 95% : 5%. As shown in Figure 10, the 95% : 5% split provides a good initialization to our model. Increasing the training set size allows the algorithm to pick a diverse initial pool from a larger training set, and hence, the model achieves a higher performance when trained on that initial pool but the performance gain does not last. Thus, we conclude that the ratio of the train-val split only has effect on the initial pool but does not show an overall improvement across AL cycles. All the other sampling strategies also follow a similar trend. The results for all the other AL sampling strategies are given in Figures S7 and S8.

## 6. Conclusion and Future Work

In this paper, we have studied the efficiency of uncertainty-based AL strategies extensively on three plant organ segmentation datasets. Our experimental results show that AL struggles to outperform random sampling in two out of three datasets we studied. We suspect that the traditional uncertainty estimation at image-level is poor by design for binary class imbalanced segmentation datasets. We believe there is a need for AL methods particularly for plant phenotyping tasks that operate at region-level where only important regions of images are sampled and labeled. We leave this for future work. We also studied how AL methods performed under differing training configurations. While we observed small improvements in AL performance with changes in data augmentations, initial pool size, AL batch size, and validation set size, we found that performing AL at smaller (even 75% smaller) image scales yields largely similar labeled datasets and ultimately similar model performances compared to operating in original image scales, proving to be a great way of cutting computation and annotation costs.

## Figures and Tables

**Algorithm 1:**
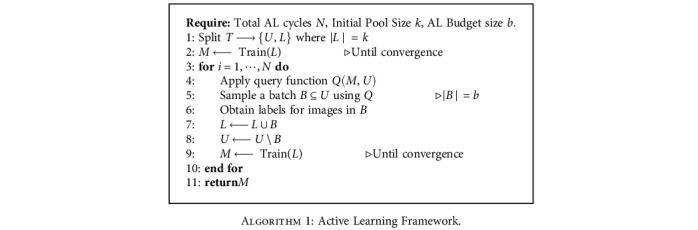
Active Learning Framework.

**Table 1:** Effect of data augmentations on model performances.

Dataset	Active learning strategies				
	RAND	LC	MAR	ENT	DBAL
Apple	+0.1%	+0.26%	-0.26%	+0.66%	-0.16%
Wheat	+1.16%	+1.30%	+1.23%	+1.43%	+1.16%
Rice	+0.13%	0.0%	+0.1%	+0.1%	+0.1%

## Data Availability

ACFR Fruit Dataset is already public. Upon acceptance of the paper, Wheat and Rice datasets with their corresponding segmentation maps will be made publicly accessible. All the PyTorch code used for experiments in this study is also public: https://github.com/ShivanganaRawat/ALPO_Segmentation. For simplicity, dataset download links will be specified in the above repository.

## Supplementary Materials

Supplementary Materials The supplementary material contains class-imbalance statistics and more results of experiments done as part of Section 5.2.
