# Supplementary material for: How Useful Is Image-Based Active Learning for Plant Organ Segmentation?
Source: Plant Phenomics. 2022 Feb 24;2022:9795275. doi: 10.34133/2022/9795275 (PMC8897744; doi:10.34133/2022/9795275)
Supplement: Supplementary Materials — The supplementary material contains class-imbalance statistics and more results of experiments done as part of Section 5.2. [file 9795275.f1.zip › Supplementary.pdf]

Supplementary Section: How Useful is Image-Based Active Learning for Plant Organ Segmentation?

immediate

1 Dataset Class Imbalance

Plant phenotying segmentation datasets have less classes as compared to standard vision segmentation datasets. In segmentation datasets each and every pixel is assigned a class label. A lot of pixels in the plant phenotying datasets belong to the background class. This leads to a heavy class imbalance in the dataset which is shown in Figure S1. The imbalance makes the training of models difficult.

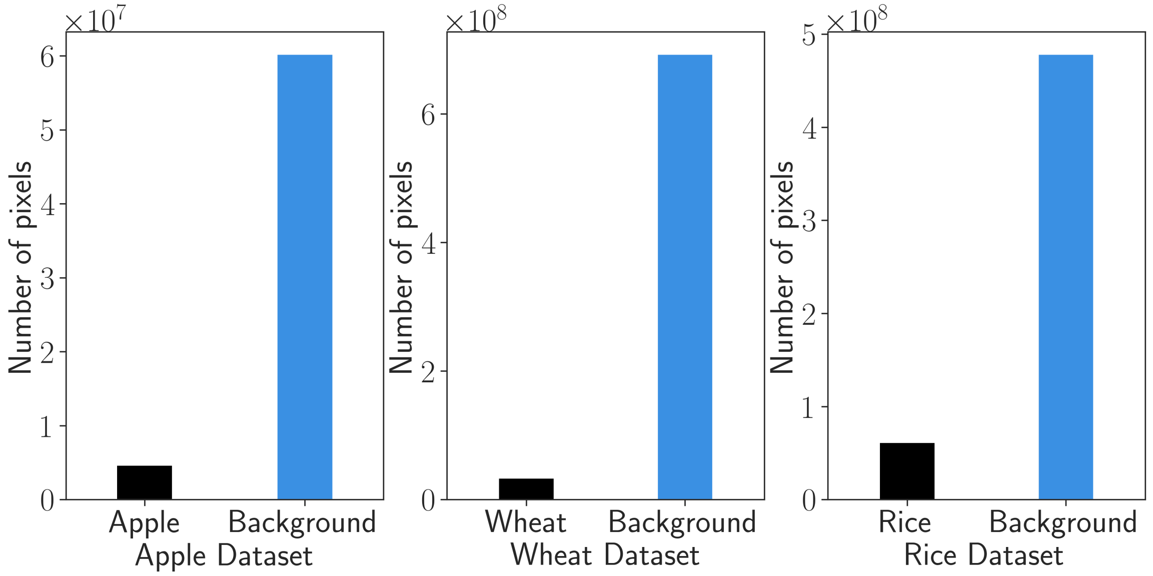

Figure S1: Dataset Class Imbalance

2 Additional Results

2.1 Active Sample Sets Overlap

Figure S2 and S3 show overlap between active sample sets picked by different AL methods on Apple and Rice datasets respectively.

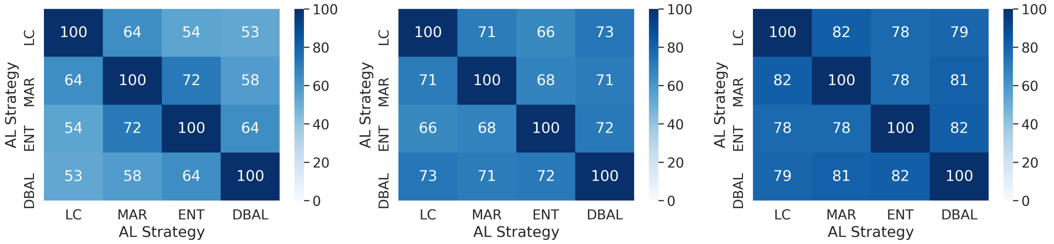

Figure S2: Overlap of active samples sets picked different AL strategies on Apple dataset

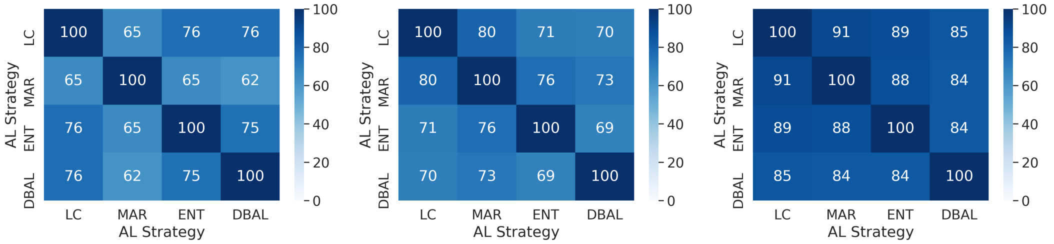

Figure S3: Overlap of active samples sets picked different AL strategies on Rice dataset

2.2 Image Scale

Figure S4 and S5 show overlap between active sample sets picked by ENT models trained on different image scales on Apple and Rice datasets respectively.

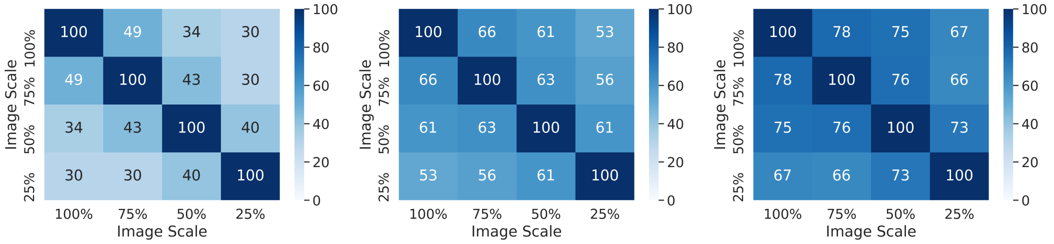

Figure S4: Overlap between active sample sets picked by ENT models trained on different image scales (Apple).

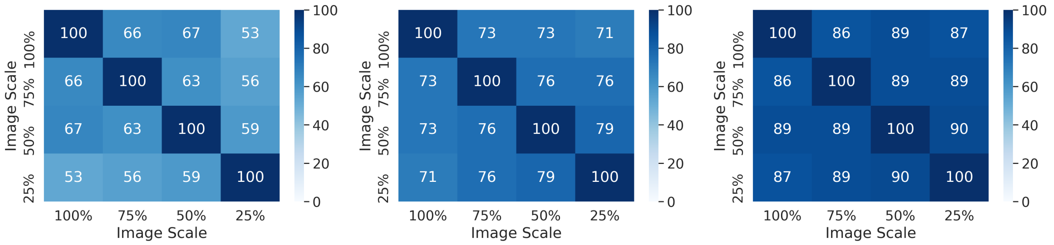

Figure S5: Overlap between active sample sets picked by ENT models trained on different image scales (Rice).

2.3 Initial Pool Size

Figure S6 shows the plots for initial pool sizes of 50, 100 and 150 for LC, MAR and DBAL for Apple, Wheat and Rice datasets.

2.4 Effect of Batch Size

Figure S7 shows the plots for batch sizes of 50, 100 and 150 for LC, MAR and DBAL for Apple, Wheat and Rice datasets.

2.5 Validation Set Size

Figure S8 shows the plots for the varying validation set size for LC, MAR and DBAL for Apple, Wheat and Rice datasets.

2.6 Train Validation Split Ratio

Figure S9 shows the plots for the varying Train Validation splits for LC, MAR and DBAL for Apple, Wheat and Rice datasets.

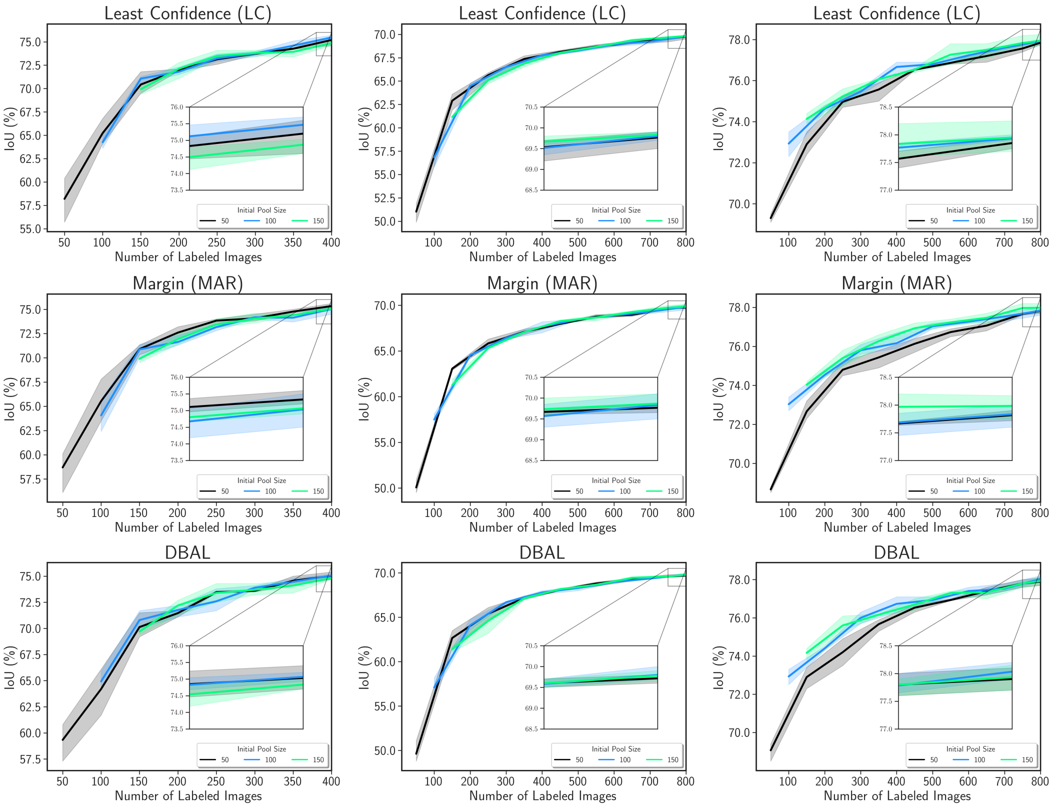

Figure S6: Effect of Initial Pool Size on Active Learning strategies. Columns 1,2 and 3 show dataset Apple, Wheat and Rice respectively.

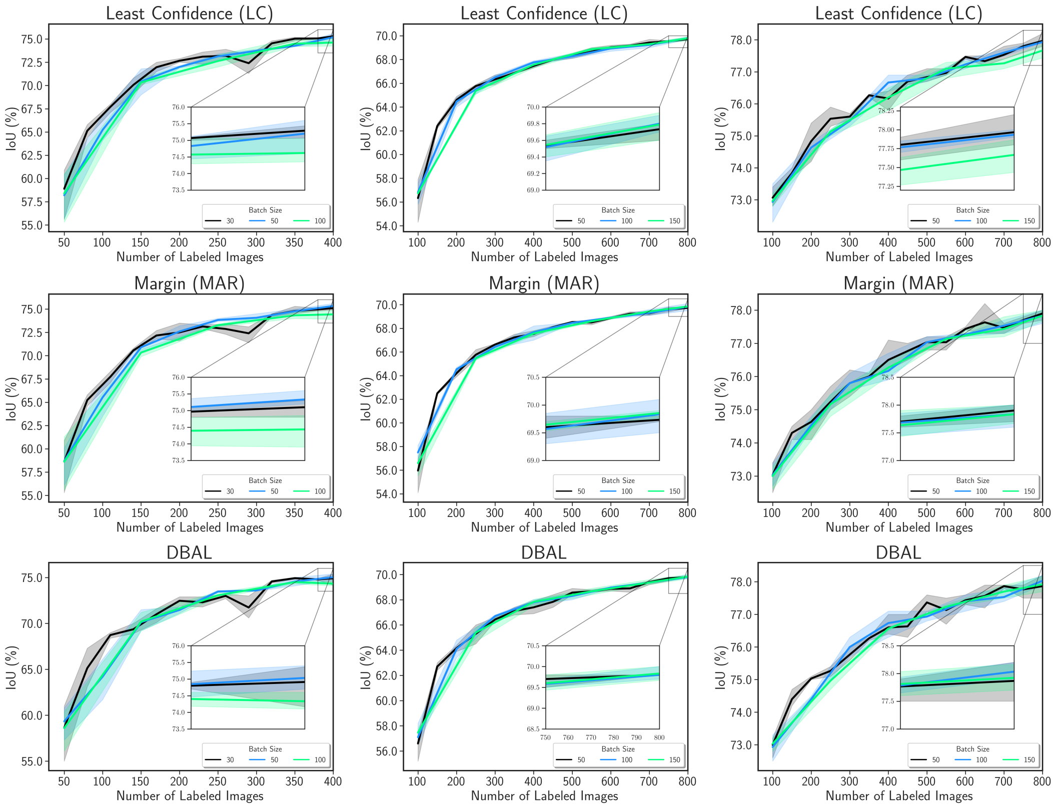

Figure S7: Effect of Batch Size on Active Learning strategies. Columns 1,2 and 3 show dataset Apple, Wheat and Rice respectively.

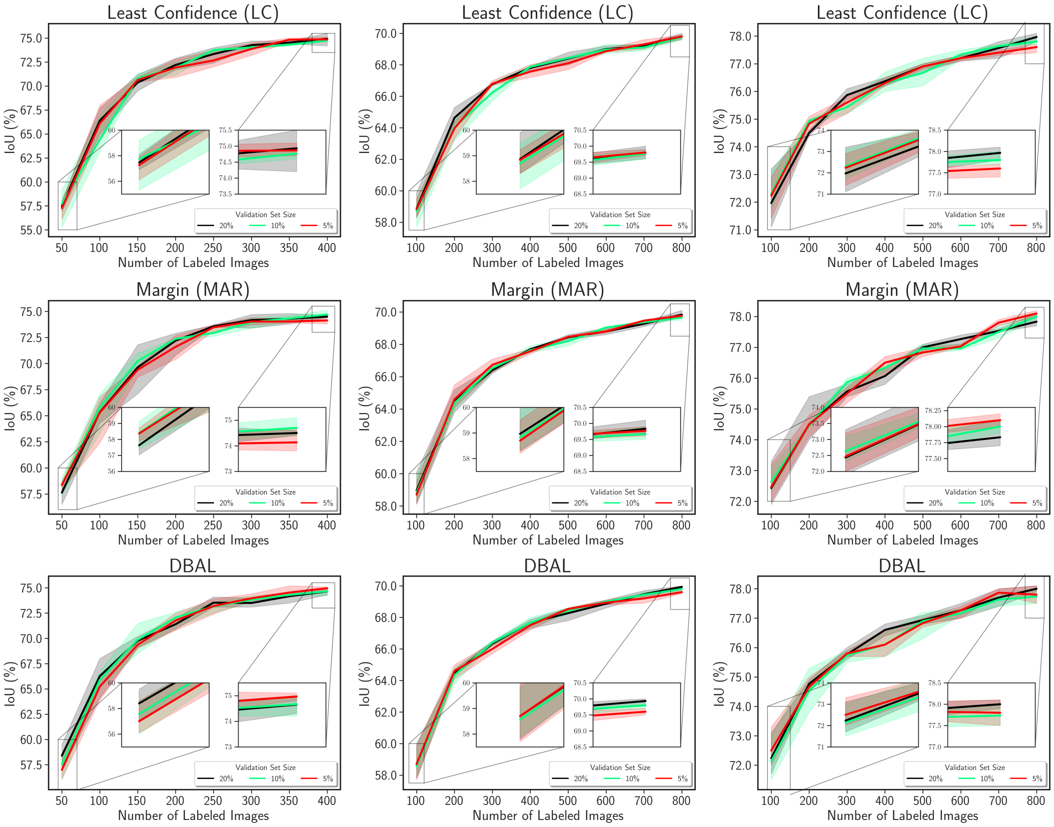

Figure S8: Effect of Validation set size on Active Learning strategies. Columns 1,2 and 3 show dataset Apple, Wheat and Rice respectively.

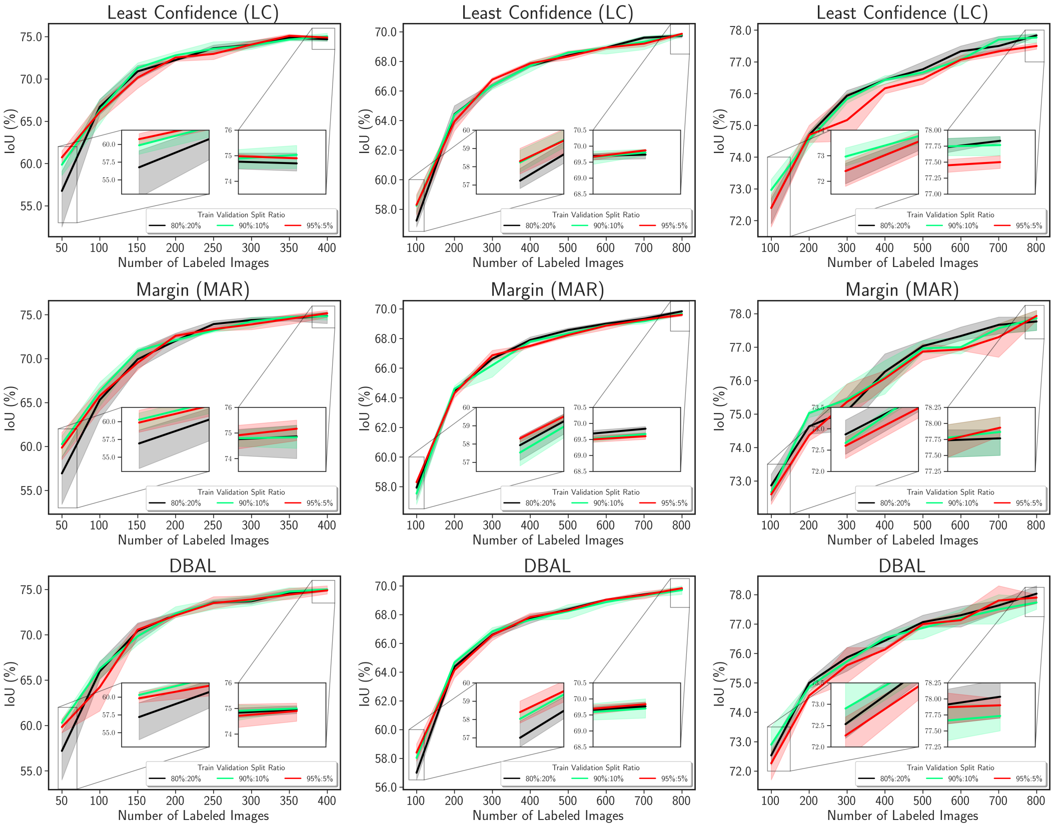

Figure S9: Effect of Train-Validation set split on Active Learning strategies. Columns 1,2 and 3 show dataset Apple, Wheat and Rice respectively.
